# Unraveling the Lipolytic Activity of Thermophilic Bacteria Isolated from a Volcanic Environment

**DOI:** 10.1155/2013/703130

**Published:** 2013-05-08

**Authors:** Panagiota M. Stathopoulou, Alexander L. Savvides, Amalia D. Karagouni, Dimitris G. Hatzinikolaou

**Affiliations:** Microbiology Group, Sector of Botany, Department of Biology, National and Kapodistrian University of Athens, Zografou Campus, Zografou 15784, Attica, Greece

## Abstract

In a bioprospecting effort towards novel thermostable lipases, we assessed the lipolytic profile of 101 bacterial strains isolated from the volcanic area of Santorini, Aegean Sea, Greece. Screening of lipase activity was performed both in agar plates and liquid cultures using olive oil as carbon source. Significant differences were observed between the two screening methods with no clear correlation between them. While the percentage of lipase producing strains identified in agar plates was only 17%, lipolytic activity in liquid culture supernatants was detected for 74% of them. Nine strains exhibiting elevated extracellular lipase activities were selected for lipase production and biochemical characterization. The majority of lipase producers revealed high phylogenetic similarity with *Geobacillus* species and related genera, whilst one of them was identified as *Aneurinibacillus* sp. Lipase biosynthesis strongly depended on the carbon source that supplemented the culture medium. Olive oil induced lipase production in all strains, but maximum enzyme yields for some of the strains were also obtained with Tween-80, mineral oil, and glycerol. Partially purified lipases revealed optimal activity at 70–80°C and pH 8-9. Extensive thermal stability studies revealed marked thermostability for the majority of the lipases as well as a two-step thermal deactivation pattern.

## 1. Introduction

Thermophilic microorganisms unequivocally represent a valuable source of highly thermostable enzymes, with numerous advantages towards biotechnological applications due to their overall inherent stability and high reaction rates at elevated temperatures [1, 2]. Among them, lipases (EC 3.1.1.3), the enzymes that catalyze both the synthesis and hydrolysis of long chain fatty acid esters (depending on water availability), constitute one of the most versatile and widely used biocatalytical group [3]. They are used in numerous diverse biotechnological applications ranging from biodiesel and biopolymers production to the synthesis of fine chemicals for medical, agrochemical, and cosmetic applications [3–5]. Due to this fact, novel thermostable lipases are in continuous demand for commercial applications especially in detergent, food, and pulp and paper industries [6]. As a result, several thermophilic microbial strains able to produce thermostable lipases have been isolated [7] and the corresponding enzymes have been purified either from the wild-type culture supernatants [8–10] or following cloning and expression in mesophilic hosts [11–14]. 

Volcanic areas represent ecological niches of rich metabolic diversity especially among bacteria and archaea [15–17], and the isolation of several thermophilic microorganisms has been described from such natural sources. The vast majority of these studies, though, is focused on the phylogenetic diversity of the corresponding microbial communities, and only few reports are available in the literature assessing the enzymatic potential of the microbial strains isolated either from volcanic [18] or other extreme environments [19] with culture dependent or metagenomic approaches. 

Pursuant to the above mentioned statements, screening of thermophilic microorganisms for lipolytic activities could facilitate the discovery, for industrial purposes, of novel lipases that are stable and function optimally at high temperatures. In this work, we examined the enzymatic lipolytic potential of 101 bacterial strains which had previously been isolated from the volcanic habitat of the Santorini volcano, at South Aegean Sea [20]. A thorough biochemical screening for extracellular lipase activity was performed both in solid and liquid media in an attempt to establish benchmark criteria for such an analysis. The most active strains were selected and characterized with respect to their 16S rDNA sequences and the effect of various carbon sources on the production of extracellular lipolytic activity. Finally, the pH and temperature-activity optima as well as the corresponding thermal stability kinetics were determined for the partially purified and concentrated lipases from all selected isolates. 

## 2. Materials and Methods

### 2.1. Microbial Strains

The 101 thermophilic bacterial strains used in this study have been previously isolated from the seawater and sediment near the active volcano of Santorini island at Aegean Sea, Greece [20]. All isolates were preserved in 30% (w/v) sterile glycerol solution at −80°C.

### 2.2. Growth Media and Conditions

All isolates were preliminary screened for their lipolytic activity both in solid and liquid cultures. A standard inoculum was prepared for each strain from glycerol stocks on Nutrient Agar (Nutrient broth plus 3% w/v agar) plates. Following incubation for 24 h at 60°C, bacterial biomass collected in sterile water and diluted to an OD_600_ of 0.3 was served as inoculum.

Solid cultures were conducted in sterile 12-well plates filled with Rhodamine B—Olive Oil agar medium containing 10 g·L^−1^ olive oil (ROA) [8]. Twenty *μ*L of standard inoculum was placed in the middle of each well (3 wells per strain), and the plates were incubated at 60°C up to 48 hours. Lipase production was evaluated from the diameter of the orange fluorescent halo (reaction of liberated fatty acids with Rhodamine B) observed under UV exposure [21]. 

Liquid cultures were conducted in 100 mL Erlenmeyer flasks with 20 mL working volume. The basal medium used consisted of (g·L^−1^): NaNO_3_, 3; K_2_HPO_4_, 1; KCl, 0.5; CaCl_2_, 0.1; MgSO_4_·7H_2_O, 0.5; yeast extract, 1; trace elements solution, 1 mL·L^−1^, supplemented with 20 g·L^−1^ olive oil as sole carbon and energy source. Triplicate flasks were inoculated for each strain, with 0.5 mL standard inoculum, and all flasks were incubated in a thermostated orbital shaker (SANYO Biomedical, UK) at 60°C and 180 rpm. Evaporation was daily checked gravimetrically and compensated with the addition of sterile water. Samples were collected every 24 h for the determination of biomass concentration and extracellular lipolytic activity. The effect of other carbon sources on lipase production was determined in a similar manner, by substituting olive oil with either glucose, mineral oil, corn cob, wheat bran, eicosane, Tween-80, or glycerol at an initial concentration of 20 g·L^−1^. Lipase production was followed by assaying of the lipase activity in the supernatant as described in enzyme assays. All lipolytic activities were determined in triplicate and reported as averages.

### 2.3. Molecular Characterization of Isolates

Bacterial identification of the nine selected isolates was based on 16S rDNA sequence analysis. Genomic DNA extraction from liquid cultures was performed according to Haught et al. [22], and the quantity of the isolated DNA was determined photometrically [23]. The 16S rDNA was amplified by polymerase chain reaction (PCR) using two universal primers [24, 25]:

 pA (5′-AGA GTT TGA TCC TGG CTC AG-3′) and
 R1492 (5′-TAC GGY TAC CTT GTT ACG ACT T-3′).

Amplification reactions were performed in volumes of 50 *μ*L containing 40 ng template DNA, 0.4 *μ*M of each primer, 1X buffer with Mg^2+^, 1 U KAPA Taq DNA polymerase (Kapa Biosystems, Woburn, MA, USA), and 0.2 mM dNTPs. Nucleases free water was used to bring the reaction volume to 50 *μ*L. After initial denaturation at 95°C for 2 min, samples were cycled for 30 PCR cycles using the following cycle profile: 95°C denaturation for 30 s, primer annealing at 53°C for 30 s, and primer extension at 72°C for 2 min, plus a final 2 min elongation step at 72°C. Amplified PCR products were separated by gel electrophoresis on 1.2% (w/v) agarose gel and then purified using NucleoSpin Extract PCR kit (MACHEREY-NAGEL, Germany). The 16S rDNA fragment (>1400 bp) was fully sequenced in both directions (Macrogen, Republic of Korea). and the corresponding gene sequences have been submitted to the GenBank databases under accession numbers: JQ808132 to JQ808140 (http://www.ncbi.nlm.nih.gov/). The 16S sequences were used for the determination of the evolutionary history (phylogenetic tree) of the nine isolates using the Neighbor-Joining method [26] in MEGA5 software [27]. The percentage of replicate trees in which the associated taxa clustered together was calculated by the bootstrap test (1000 replicates) [28]. Evolutionary distances were computed using the Maximum Composite Likelihood method [29]. 

### 2.4. Preparation of Partially Purified Lipase Samples

Liquid cultures of the nine selected strains were conducted in 2 L Erlenmeyer flasks of 400 mL working volume (3 flasks per strain) as described above. Biomass was removed by centrifugation (6000 rpm, 5 min), and the resulting supernatant was passed through Whatman number 1 filter paper in order to remove the residual olive oil. The filtrate was then subjected to ammonium sulphate precipitation. Optimum precipitation range was determined by employing a gradual ammonium sulphate procedure (5% saturation step) and measuring specific lipase activity on the resulting supernatants and precipitates. The optimum precipitation conditions were subsequently applied to each culture supernatant and the collected precipitates were resuspended in 20 mM potassium phosphate buffer (pH 8) and desalted using PD-10 gel filtration columns (GE Healthcare) using the above buffer for elution. The concentrated and desalted enzyme solutions were used for the determination of the optimum pH and temperature and thermal stability of the corresponding lipolytic activities. 

### 2.5. Enzyme Assays

Lipase activity assays in liquid samples were routinely performed using p-nitro phenyl palmitate (pNPP) as substrate in 50 mM Tris-HCl buffer, pH 8, essentially as described by Vorderwülbecke et al. [30] with minor modifications. pNPP was dissolved in propane-2-ol at a final concentration of 7.5 mg·mL^−1^. The final substrate solution (950 *μ*L) was prepared by dropwise addition, under continuous vortexing, of 95 *μ*L pNPP solution into 855 *μ*L of buffer solution (prepared by dissolving 0.4 g of Triton X-100 and 0.1 g gum arabic in 90 mL of buffer). The reaction was initiated by adding 50 *μ*L sample into the above substrate solution followed by incubation in a water bath at 60°C for 15 min. The reaction was terminated by placing the reaction mixture on ice for 10 min. p-nitro phenol (pNP) concentration was determined by measuring the absorbance at 410 nm using a calibration curve constructed at the assay buffer (pH 8). For blank, we used heat sterilized (15 min, 121°C) samples undergone the same procedure. Activity was expressed as nkat·mL^−1^.

### 2.6. Determination of Lipase Thermal Stability and Activity Optima

Optimum temperature for lipase activity was determined under the above described assay conditions at pH 8, using an incubation temperature range from 50 to 100°C. Optimum pH values were determined in the same manner at 60°C using an assay buffer range from 6 to 10. A different calibration curve was constructed and used for pNP at each pH value used. For temperature stability, enzyme samples were incubated in sealed screw-cap vials, at pH 7 (phosphate buffer, 100 mM) at various temperatures, sample aliquots were withdrawn periodically, and remaining activity was determined at regular assay conditions. All enzyme activities were determined in triplicate and reported as averages.

## 3. Results and Discussion

### 3.1. Detection of Lipolytic Activity

The lipolytic activities of all isolates were assessed both qualitatively (agar plate cultures) and quantitatively (liquid cultures) using olive oil as sole carbon and energy source. The corresponding results are summarized in Table 1. Only 17 out of 101 (16.8%) strains produced clear fluorescent halos, designating free fatty acid release, during growth on ROA plates. On the contrary, in liquid cultures significant lipolytic activity (>0.05 nkat/mL) was detected in the culture supernatant of 56 strains (55.4%) while for 19 strains (18.9%) the corresponding average activities were very low (<0.05 nkat/mL) but detectable. For 26 strains (25.7%) no activity was detected. No clear correlation could be identified among the two screening methods used. As a result strains such as SP6 and SP71 yielded clear fluorescent halos during growth on plates while the same strains produced only low levels of lipase in liquid cultures; in addition strains like SP14, SP22, SP29, SP76, and SP79 revealed the opposite lipolytic phenotype (no halo on ROA, high extracellular lipolytic activity in OLM). Only four out of the 101 strains, namely, SP73, SP75, SP83, and SP93, revealed an equivalent lipolytic profile both on solid and liquid cultures. In addition, we could not descry a single strain yielding fluorescence halos on Rhodamine B agar plates without displaying even a small amount of extracellular lipolytic activity in liquid cultures. 

The Rhodamine B plate assay has been widely used in the past by many researchers as the sole screening method for the identification of lipase producing microorganisms from various environmental samples [31], including extreme environments such as hot springs and soda lakes [32–34]. Our results clearly demonstrate the fact that the solid-phase Rhodamine system has failed to detect all the lipase producing microorganisms even though olive oil (a true lipase inducer) was employed as sole carbon and energy source. Liquid cultures on olive oil combined with an extracellular enzyme assay proved more reliable in identifying the lipolytic potential of the strains under consideration. The lower sensitivity of Rhodamine assay that is sometimes used to explain the discrepancy between the two approaches can be easily overruled in our case, by the fact that we were able to identify strains that yielded very clear halos with only traces of lipase activity in liquid cultures. These results clearly show that liquid culture screening on various lipase inducers yields more reliable results and should be preferred, especially today, where several high throughput liquid enzymatic assay screening methods are being available [35, 36].

### 3.2. Phylogeny of Lipase Producers

**Figure 1:**
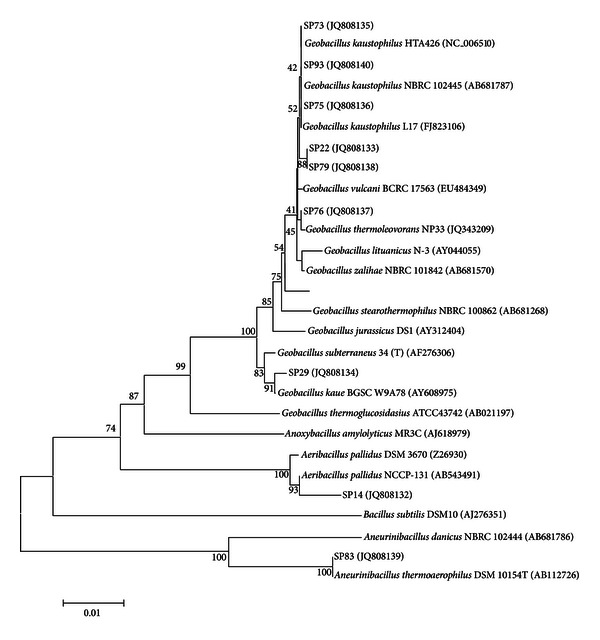
Evolutionary relationships of the selected strains using the Neighbor-Joining method. The bootstrap consensus tree was inferred from 1000 replicates. Branches corresponding to partitions reproduced in less than 50% bootstrap replicates are collapsed. The percentage of replicate trees in which the associated taxa clustered together in the bootstrap test (1000 replicates) is shown next to the branches. The tree is drawn to scale, with branch lengths in the same units as those of the evolutionary distances used to infer the phylogenetic tree. The evolutionary distances were computed using the Maximum Composite Likelihood method and are in the units of the number of base substitutions per site. All positions containing gaps and missing data were eliminated.

Following the results of this primary screening, we decided to proceed with our work by selecting those strains that either excreted high average extracellular lipolytic activity in liquid cultures (>0.75 nkat·mL^−1^) or produced intense fluorescent halos in Rhodamine media. The nine strains that fulfill these criteria are in bold italics in Table 1. The selected strains were subjected to 16S rDNA sequencing (NCBI Accession numbers: JQ808132 to JQ808140) and phylogenetic analysis. A phylogenetic tree constructed using the corresponding sequences along with those from annotated *Geobacillus *and other related genera is given in Figure 1. All nine lipolytic strains revealed great phylogenetic similarity (>99%) with known *Geobacillus* and other closely related species. This high 16S rDNA similarity is characteristic of this relatively recently characterized genus [37] that includes species with promising biotechnological potential [38]. Members of geobacilli have been reported to represent the majority of the species that inhabit a variety of diverse “hot” environments such as marine thermal vents [39, 40], high temperature oil fields [37], hot springs [41], geothermal regions [42], and sugar refinery wastewaters [43]. 

In our case, three of the strains, namely, SP73, SP75, and SP93 have been clustered together with known *Geobacillus kaustophilus* strains most probably representing strains of this species. To our knowledge there are no published reports on lipolytic activities from *G. kaustophilus* related strains although it was possible to identify a provisional putative lipase gene in the *G. kaustophilus* HTA426 complete genome sequence (NCBI Accession number NC_006510, Gene ID: 3185865). Isolates SP22 and SP79 that revealed identical 16S rDNA partial sequences clustered together with close phylogenetic similarity to the *G. kaustophilus *group, while isolates SP76 and SP29 can clearly be characterized as members of the *Geobacillus *group closely related to *G. thermoleovorans *and *G. kaue*, respectively. *G. thermoleovorans* is a known lipase producer among the geobacilli, and the corresponding enzymes from various strains of this species have been purified and characterized either from wild-type cultures [9, 44] or following heterologous expression in *E. coli* [12, 45, 46]. On the contrary, there is no information in the literature concerning lipolytic activity by *G. kaue *strains. Isolate SP14 clustered together with the recently characterized *Aeribacillus pallidus* species, the only characterized member in the *Aeribacillus* taxonomic group [47]. Concerning lipase production, only an *A. pallidus* strain isolated from thermal sites in Mexico, has been reported to express lipolytic activity in ROA plates [48], although this was not the case for strain SP14 that showed high extracellular lipase activity only in liquid cultures. Finally, strain SP83 revealed an identical 16S rDNA sequence with *Aneurinibacillus thermoaerophilus* DSM 10154, representing the most phylogenetically distant member among the lipase producers in our study. The *Aneurinibacillus* genus does not belong to the Bacillaceae family (like *Geobacillus* and *Aeribacillus*), since is a member of the Paenibacillaceae family [49]. Strains related to this group have been isolated from geothermal soils throughout the globe [50, 51], while lipase production has been optimized in submerged cultures of an *A. thermoaerophilus* strain isolated from a Malaysian hot spring [52].

### 3.3. Lipase Production Pattern

**Figure 2:**
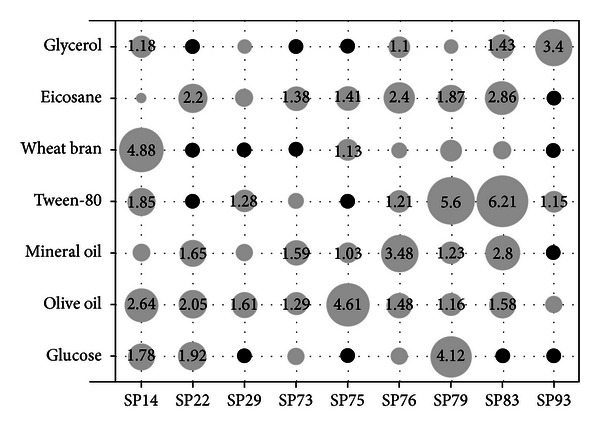
Effect of carbon source type on maximum extracellular lipase production (nkat·mL^−1^) in liquid cultures, achieved between 48 and 72 h of growth, depending on the source used. Circle areas are proportional to enzyme activities, while only activities above 1 nkat·mL^−1^ are shown with numerical values. Black circles denote no detection of lipolytic activity. Values represent the mean of triplicate flasks. Maximum SD ± 18.2%.

The effect of carbon source on lipase production by the nine selected isolates was studied using the minimal basal medium supplemented with an initial concentration of 20 g·L^−1^ of various carbon sources. Samples were withdrawn twice a day, and the lipase activity was determined in the cell free supernatant. Growth of the microorganisms was observed in all cases as verified by either OD_600_ or cfu measurements (data not shown).  Figure 2 summarizes the corresponding lipase production pattern which appeared to be quite diverse among the nine isolates. Olive oil, a well-known lipase inducer, was able to induce significant lipase production levels in all nine strains, although it proved to be the optimal carbon source only for strain SP75. These results justify its use as the preferred carbon source in various screening studies for lipolytic activity in thermophilic bacteria [7] or in studies concerning the optimization of lipase production using wild type strains [53]. Tween-80, an oleic acid ester (Polyoxyethylene sorbitan monooleate), yielded maximum lipase titers in strains SP79 and SP83, but it completely inhibited lipase production in strains SP22 and SP75. Its addition in culture media for lipase production by members of the Bacillaceae family has often been evaluated, exhibiting either inducing [54–56] or inhibiting effect [8, 53, 57, 58]. Optimum lipase production with Tween-80 as sole carbon source has been also achieved with a *G. stearothermophilus* strain following medium optimization [59]. All but one strains were able to produce at least some lipase activity in the presence of pure alkanes (eicosane) or alkane mixtures (mineral oil) as sole carbon sources. Stimulation of lipase production by aliphatic hydrocarbons in bacteria, although documented several decades ago [60], has been reported only once for members of the Bacillaceae family [44]. It is also noteworthy that eicosane and mineral oil revealed very similar lipase production patterns among the nine strains (Figure 2), suggesting that lipase biosynthesis by alkanes in thermophilic bacteria is induced in a manner independent of their chain length. 

The two low molecular weight compounds examined, glycerol and glucose, showed limited lipase induction ability with two notable exceptions of an apparent inducing effect; that of glycerol for strain SP93 and that of glucose for strain SP79. A repressing effect of glucose [55, 61] or glycerol [62] on lipase production by members of the Bacillaceae family has been reported in several studies, but this does not seem to be a universal phenomenon, since both glucose and glycerol were found to be excellent carbon sources for lipase production by the *G. stearothermophilus *strain-5 [59, 63]. Wheat bran, a complex agricultural byproduct, was also examined on the basis of its use in several reports as an inducer of lipase activity by various fungi [64, 65], despite the fact that there are no references concerning its use as carbon source for lipase production in bacteria. Wheat bran was indeed a poor lipase inducer for the majority of the bacterial strains, but quite surprisingly it was proved to be the optimal carbon source for the *Aeribacillus *sp. strain SP14. The latter isolate was also among the three strains that were able to produce lipase in all carbon sources examined, the other two being strains SP76 and SP79.

Lipase production among the nine isolates did not reveal any specific correlations with their evolutionary relationships. Phylogenetically close isolates such as SP22 and SP79 showed quite diverse lipolytic activity patterns, while the opposite was observed with several other pairs of isolates such as SP14 and SP79. The only weak relation involved the three *G. kaustophilus* strains of our study (SP73, SP75, and SP93) that proved to be the poorest lipase producers, both in substrate diversity and maximum observed lipolytic activities. It is noteworthy though to point out that the maximum lipase activities obtained in this experimental set are comparable to those reported in studies concerning the optimization of medium/culture parameters for lipase production by various thermophilic *Bacillus *and *Geobacillus *strains [53], despite the fact that we did not perform any optimization efforts. 

### 3.4. Assessment of Lipolytic Activities

**Figure 3:**
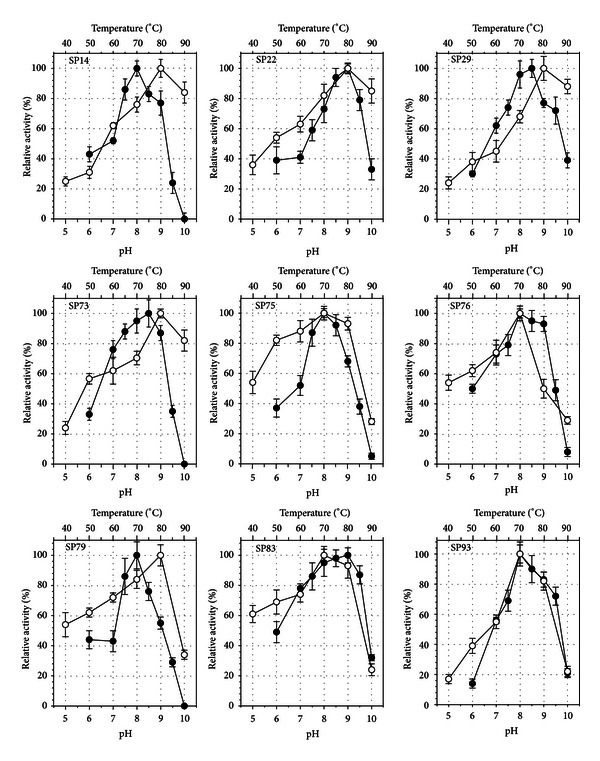
Effect of pH (*●*) and temperature (○) on the activity of the partially purified lipases from the nine selected strains. Error bars represent the SD from triplicate determinations.

**Figure 4:**
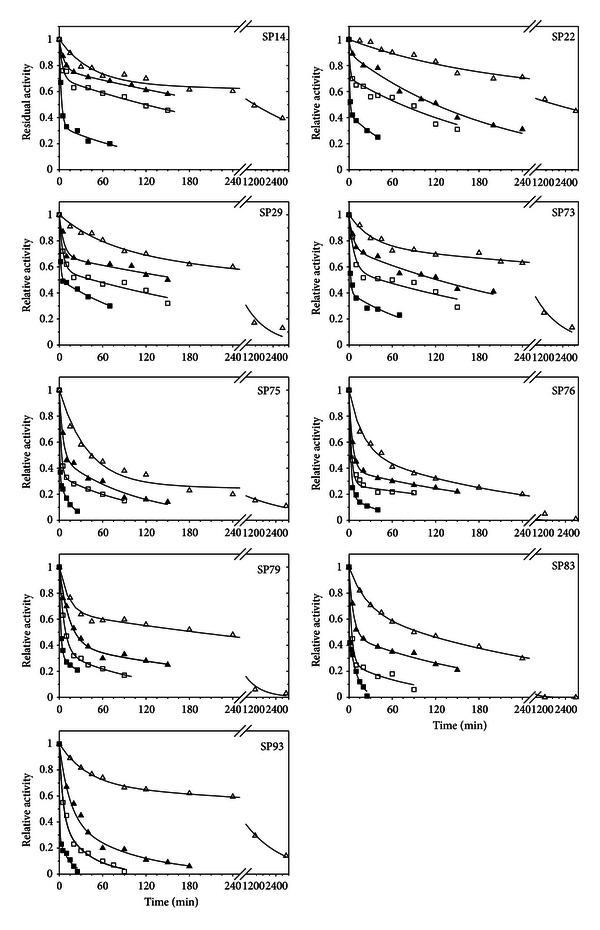
Thermal stability profiles of the nine partially purified lipases. ∆: 70°C; ▲: 80°C; □: 90°C; ■: 100°C. Straight lines represent the non-linear fitting of (2) in the experimental data.

Following partial purification from the corresponding culture supernatants, we performed a biochemical characterization of the nine lipases by determining their temperature and pH-activity optima (Figure 3) as well as their thermostability kinetics (Figure 4). All lipases had a relatively basic pH optimum between 8 and 9. The functional pH range was relatively narrow between 7 and 9.5 with most of the enzymes showing a sharp decrease in the activity at pH 10. The lipases from strains SP22 and SP83 were the most alkalophilic, showing optimum activity at pH 9 and maintaining more than 30% of their activity at pH 10. These pH optima are similar to those reported for several *Bacillus *species [8, 11, 66, 67], although the pH-activity profiles for the majority of mesophilic *Bacilli *are wider, probably because pH effects are being determined at moderate temperatures between 20 and 30°C. As far as the lipases from other geobacilli are concerned, the reported pH optima are also 8 [32, 63, 68] or 9 [12, 13, 69, 70] indicating a universal lipolytic pH-activity pattern within the genus.

 The temperature optima determined for the nine partially purified enzymes were relatively high and reached the level of either 70 (4 isolates) or 80°C (5 isolates). The lipases for strains SP14 (*A. pallidus*), SP22, SP73, SP79 (*G. kaustophilus*), and SP29 (*G. kaue*) had the highest temperature-activity optima maintaining (with the exception of isolate SP79) more than 80% of their optimum activity even at the temperature of 90°C. These lipolytic activity optima are significantly higher than those reported for other geobacilli. More specifically, the optimum reported activity temperatures were 55°C for the *G. stearothermophilus* JC [13] and *Geobacillus* sp. TW1 [32] lipases, 60°C for *Geobacillus* sp. SBS-4S [69] and *G. thermoleovorans* CCR11 [12], 65°C for *Geobacillus* sp. strain ARM [68], G. *stearothermophilus *strain-5 [63], and *G. thermoleovorans* [45, 71] and 70°C for the lipase of *Geobacillus* sp. T1 [70], and *G. zalihae* [7]. These results indicate that the Santorini volcanic habitat is most probably an environment that hosts bacterial species that harbor enzymatic activities of exceptional thermophilicity.

 The above promising results concerning the temperature optima of the nine partially purified lipases led us to pursue a thorough study on their thermal stability properties in 10°C increments. In agreement with their high optimum temperatures, the lipases from the Santorini geobacilli revealed very significant thermal stability properties. With the exception of the lipases from isolates SP75 and SP76 that maintained 82% and 75% of their activity, respectively, after 1 h incubation at 60°C all other lipases maintained over 90% of their activity at this temperature and incubation time (data not shown). For the temperature range from 70 to 100°C, thermal deactivation curves are given in Figure 4. Analysis of the time point data for all enzymes using SigmaPlot software revealed that first order deactivation was not able to adequately describe the deactivation kinetics of the lipases at various temperatures. In an effort to find the best fit model we applied the approach of Henley and Sadana [72] where lipase thermal inactivation is sought to take place following a two-step deactivation process, through an intermediate state, as follows:

(1) E0→k1E1→k2E2,

where *E*_0_, *E*_1_, and *E*_2_ are the specific activities of the initial, intermediate, and final state, respectively, and *k*_1_ and *k*_2_ are first-order deactivation constants. By defining *α*_1_ = *E*_1_/*E*_0_ and *α*_2_ = *E*_2_/*E*_0_ and assuming that the *E*_2_ state is completely inactive (*a*_2_ = 0), the observed residual enzyme activity as a function of time, *α*, is given by (2) following integration over time [72]:

(2) α=[1+α1k1k2−k1]·exp⁡(−k1t)  −[a1k1k2−k1]·exp⁡(−k2t).

The fitting of the experimental data into (2) for each lipase and temperature was performed using the nonlinear regression routines of Sigmaplot software, and the results are presented in Figure 4 and Table 2. Regression was allowed to converge freely into the least-squares value and the only restriction that was applied concerned the *α*_1_ specific activity ratio that was confined between the values of 0 and 1. 

All lipases seemed to follow the two-step deactivation scheme, since we observed an excellent fitting of the experimental data into (2). This means that the enzymes upon the effect of temperature undergo a relatively rapid inactivation into the *E*_1_ intermediate form, which has lower activity compared to the native enzyme (*E*_0_). The deactivation rate, though, from the intermediate form to the completely inactive enzyme (*E*_2_) is significantly slower, advocating for a more compact and heat resistant character for the *E*_1_ enzyme. This was verified by the fact that for all enzymes and at all temperatures examined the values of the deactivation constant *k*_2_ were on average two orders of magnitude lower than the corresponding *k*_1_ levels (Table 2). With the exceptions of the lipases from isolates SP75 and SP76 above mentioned, all other enzymes showed exceptional thermal stabilities that allowed clear detection of activity even after 1 h incubation at 100°C. The most thermostable enzymes were those of strains SP14, SP22, SP29, and SP73. The most significant differentiation of these four lipases that seemed to contribute in their observed high thermal stability was the fact that the specific activity ratio *α*_1_ for these enzymes was markedly higher than the other five lipases, and more importantly, its value was practically stable in the temperature range from 70 to 90°C. As was the case with their temperature-activity optima, the lipases from the Santorini thermophilic bacilli were also more thermostable than the majority of other *Geobacillus* sp. lipases reported in the literature. Although this is the first extensive study on the thermal stabilities for *Geobacillus* lipases, literature data fully support our claim, since complete loss of activity has been reported for the *G. zalihae* lipase following 30 min incubation at 80°C [7], the *Geobacillus* sp. SBS-4S lipase when exposed for 100 min at 60°C [69], and the lipase from *G. stearothermophilus* JC after 30 min at 70°C [13], while the *Geobacillus* sp. strain ARM lipase had half lives of only few minutes at 60 and 70°C [68].

## 4. Conclusions

An extensive assessment of the lipolytic potential in a set of thermophilic bacteria isolated from the Santorini volcanic habitat was performed. By combining both solid and liquid culture techniques, a widespread lipolytic phenotype of variable intensity was revealed, that involved almost 75% of the isolates. The genus *Geobacillus* was the dominant one within the subset of nine strains selected for their high extracellular lipase production. Enzyme levels were strongly dependent on the type of carbon source, but we were not able to clearly identify a universal phenotype for lipase induction among the strains. The biochemical characterization of the nine lipases marked a significant differentiation to most other *Geobacillus* lipases with respect to the effect of temperature on the activity and stability of the corresponding enzymes. The majority of lipases from the Santorini thermophilic bacteria revealed exceptional thermostability with high optimum activity temperatures, thus representing very promising candidate enzymes for a variety of high temperature industrial lipolytic applications. Such an endeavor would probably require their efficient cloning and overexpression in mesophilic hosts, even though that for some of the strains lipase production was at relatively high levels compared to other wild-type thermophilic bacterial strains.

## Figures and Tables

**Table 1:** Comparison of the lipolytic activities among the 101 thermophilic bacterial isolates in Rhodamine olive oil agar (ROA) and olive oil liquid (OLM) cultures.

Strain	ROA		OLM	
	24 h	48 h	48 h	72 h
SP1	−	−	−	−
SP2	−	−	0.040	0.063
SP3	−	−	−	−
SP4	−	+	0.062	0.102
SP5	−	−	−	−
SP6	+	+	0.058	0.073
SP7	−	−	0.250	0.085
SP8	−	−	0.250	0.270
SP9	−	−	0.307	0.167
SP10	−	−	0.500	0.200
SP11	−	−	0.358	0.217
SP12	−	−	0.350	0.142
SP13	−	−	0.583	0.175
***SP14 ***	***− ***	***− ***	***1.070 ***	***2.367 ***
SP15	−	−	0.108	0.147
SP16	−	−	0.022	0.065
SP17	−	−	−	−
SP18	−	−	0.200	0.067
SP19	−	−	0.217	0.248
SP20	−	−	0.153	0.055
SP21	−	−	0.550	0.427
***SP22 ***	***− ***	***− ***	***1.618 ***	***0.450 ***
SP23	−	−	0.567	0.192
SP24	−	−	0.217	0.133
SP25	−	+	0.044	0.092
SP26	−	−	−	−
SP27	+	+	0.150	0.288
SP28	−	−	−	−
***SP29 ***	***− ***	***− ***	***0.967 ***	***0.620 ***
SP30	−	+	0.422	1.027
SP31	−	−	−	−
SP32	−	+	0.011	0.085
SP33	−	−	−	−
SP34	−	−	−	−
SP35	−	−	0.095	0.258
SP36	−	−	−	−
SP37	−	−	−	−
SP38	−	−	0.178	0.767
SP39	−	−	−	−
SP40	−	−	0.063	0.115
SP41	−	−	−	−
SP42	−	−	0.450	0.192
SP43	−	−	0.120	0.072
SP44	−	−	0.120	0.123
SP45	−	−	0.153	0.088
SP46	−	−	−	−
SP47	−	−	0.038	0.045
SP48	−	−	0.043	0.027
SP49	−	−	0.033	0.032
SP50	−	−	0.042	0.037
SP51	−	−	0.058	0.032
SP52	−	−	−	−
SP53	−	−	−	−
SP54	−	−	0.037	0.045
SP55	−	−	0.035	0.062
SP56	−	−	0.052	0.000
SP57	−	−	0.038	0.183
SP58	−	−	−	−
SP59	−	−	0.000	0.085
SP60	−	−	−	−
SP61	−	−	0.040	0.037
SP62	−	−	−	−
SP63	−	−	0.038	0.053
SP64	−	−	0.057	0.000
SP65	−	−	0.045	0.043
SP66	−	−	0.115	0.047
SP67	−	−	0.053	0.075
SP68	−	−	−	−
SP69	−	−	−	−
SP70	−	−	0.043	0.070
SP71	+	+	0.043	0.052
SP72	−	−	0.208	0.244
***SP73 ***	***+ ***	***++ ***	***1.083 ***	***0.740 ***
SP74	−	−	0.088	0.000
***SP75 ***	***+ ***	***++ ***	***1.540 ***	***1.667 ***
***SP76 ***	***− ***	***− ***	***1.297 ***	***1.317 ***
SP77	−	−	0.483	0.228
SP78	−	−	0.220	0.150
***SP79 ***	***− ***	***− ***	***1.167 ***	***1.245 ***
SP80	+	+	0.137	0.110
SP81	−	−	0.543	0.293
SP82	−	+	0.235	0.250
***SP83 ***	***+ ***	***+ ***	***0.625 ***	***1.433 ***
SP84	−	+	0.167	0.133
SP85	−	−	0.077	0.000
SP86	−	−	0.063	0.070
SP87	−	+	0.058	0.000
SP88	−	−	−	−
SP89	−	−	−	−
SP90	−	+	0.593	0.233
SP91	−	−	0.517	0.767
SP92	+	+	0.517	0.522
***SP93 ***	***++ ***	***++ ***	***0.610 ***	***0.633 ***
SP94	−	−	0.550	0.257
SP95	−	−	1.027	0.600
SP96	−	−	0.073	0.085
SP97	−	−	−	−
SP98	−	−	−	−
SP99	−	−	−	−
SP100	−	−	0.400	0.642
SP101	−	−	0.000	0.122

In ROA cultures, qualitative evaluation was performed based on fluorescence halo production (−: no halo; +: partial coverage of the plate; ++: full coverage of the plate). In OLM cultures, values represent the extracellular lipolytic activity in nkat·mL^−1^ (average of triplicate flasks: max. SD ± 9.4%). Bold/italicized lines represent those strains selected for further study.

**Table 2:** Values of the kinetic constants for the thermal deactivation kinetics as determined through nonlinear regression fitting of the experimental data in (2). Activation energies for *k*_1_ and *k*_2_ were determined from the Arrhenius equation through linear regression.

		SP14	SP22	SP29	SP73	SP75
*α*_1_	70°C	0.647 ± 0.021	0.589 ± 0.060	0.662 ± 0.055	0.740 ± 0.021	0.267 ± 0.030
	80°C	0.759 ± 0.007	0.879 ± 0.035	0.673 ± 0.040	0.726 ± 0.028	0.459 ± 0.044
	90°C	0.690 ± 0.026	0.687 ± 0.026	0.566 ± 0.031	0.562 ± 0.059	0.335 ± 0.005
	100°C	0.328 ± 0.027	0.432 ± 0.006	0.500 ± 0.011	0.399 ± 0.034	0.302 ± 0.009

*k*_1_ (min⁡^−1^)	70°C	2.24 ± 0.37 × 10^−2^	5.06 ± 1.14 × 10^−3^	1.43 ± 0.44 × 10^−2^	3.21 ± 0.74 × 10^−2^	2.54 ± 0.30 × 10^−1^
	80°C	1.54 ± 0.12 × 10^−1^	3.45 ± 0.36 × 10^−1^	1.57 ± 0.54 × 10^−1^	1.66 ± 0.47 × 10^−1^	2.07 ± 0.45 × 10^−1^
	90°C	2.30 ± 0.65 × 10^−1^	5.33 ± 0.36 × 10^−1^	2.04 ± 0.45 × 10^−1^	1.45 ± 0.52 × 10^−1^	3.96 ± 0.13 × 10^−1^
	100°C	3.59 ± 0.40 × 10^−1^	8.81 ± 0.37 × 10^−1^	6.47 ± 0.54 × 10^−1^	6.27 ± 1.27 × 10^−1^	1.42 ± 0.08 × 10^0^
	*E*_a_ (kJ/mole)	93.72	171.58	125.24	93.56	60.96

*k*_2_ (min⁡^−1^)	70°C	1.82 ± 0.27 × 10^−4^	9.42 × 1.49 × 10^−5^	8.26 ± 1.13 × 10^−4^	7.06 ± 0.62 × 10^−4^	3.59 ± 1.33 × 10^−4^
	80°C	1.83 ± 0.10 × 10^−3^	4.85 ± 0.31 × 10^−4^	1.76 ± 0.64 × 10^−3^	3.21 ± 0.35 × 10^−3^	9.20 ± 1.48 × 10^−3^
	90°C	2.81 ± 0.45 × 10^−3^	4.96 ± 0.56 × 10^−3^	3.05 ± 0.66 × 10^−3^	3.27 ± 1.22 × 10^−3^	9.09 ± 0.34 × 10^−3^
	100°C	7.84 ± 2.32 × 10^−3^	1.45 ± 0.07 × 10^−2^	7.43 ± 0.68 × 10^−3^	9.42 ± 2.68 × 10^−3^	6.19 ± 0.32 × 10^−2^
	*E*_a_ (kJ/mole)	125.29	185.85	80.47	83.19	165.09

		SP76	SP79	SP83	SP93	

*α*_1_	70°C	0.493 ± 0.055	0.639 ± 0.013	0.627 ± 0.026	0.662 ± 0.006	
	80°C	0.377 ± 0.008	0.384 ± 0.047	0.458 ± 0.029	0.380 ± 0.011	
	90°C	0.270 ± 0.048	0.330 ± 0.022	0.254 ± 0.053	0.366 ± 0.083	
	100°C	0.176 ± 0.054	0.313 ± 0.013	0.491 ± 0.052	0.271 ± 0.065	

*k*_1_ (min⁡^−1^)	70°C	5.36 ± 1.21 × 10^−2^	7.23 ± 1.63 × 10^−2^	4.12 ± 0.53 × 10^−2^	2.57 ± 0.12 × 10^−2^	
	80°C	2.02 ± 0.08 × 10^−1^	8.01 ± 1.17 × 10^−2^	1.64 ± 0.23 × 10^−1^	7.22 ± 0.23 × 10^−2^	
	90°C	2.90 ± 0.68 × 10^−1^	1.48 ± 0.10 × 10^−1^	2.77 ± 0.60 × 10^−1^	2.02 ± 0.05 × 10^−1^	
	100°C	3.68 ± 0.58 × 10^−1^	5.05 ± 0.27 × 10^−1^	1.19 ± 0.47 × 10^1^	1.87 ± 0.46 × 10^0^	
	*E*_a_ (kJ/mole)	66.00	67.92	185.06	147.11	

k_2_ (min⁡^−1^)	70°C	3.77 ± 0.82 × 10^−3^	1.39 ± 0.16 × 10^−3^	3.30 ± 0.24 × 10^−3^	5.63 ± 0.13 × 10^−4^	
	80°C	4.21 ± 0.25 × 10^−3^	3.04 ± 0.64 × 10^−3^	4.88 ± 0.80 × 10^−3^	1.09 ± 0.03 × 10^−2^	
	90°C	2.06 ± 0.35 × 10^−2^	7.81 ± 1.43 × 10^−3^	1.12 ± 0.49 × 10^−2^	2.62 ± 0.61 × 10^−2^	
	100°C	3.23 ± 1.50 × 10^−2^	1.77 ± 0.27 × 10^−2^	9.49 ± 0.88 × 10^−2^	7.25 ± 1.50 × 10^−2^	
	*E*_a_ (kJ/mole)	85.26	91.24	115.04	165.59	
